# The Story of the Insane from Year to Year

**Published:** 1901-11-23

**Authors:** 


### THE STORY OF THE INSANE FROM YEAR TO YEAR.

(Thirteenth Series.)

### LONDON COUNTY ASYLUM AT CLAYBURY.

In this asylum 2,491 patients were in residence on January 1st, 1899, and this number was reduced to 2,452 on December 31st. There were 492 first admissions, 64 readmissions, 197 recovered, 132 discharged relieved, and 265 deaths. The average number resident during the year was 2,482, and the total number of persons under treatment was 3,047. The recoveries, calculated in the usual way, stand at 39·64 per cent., and the deaths reckoned on the average number resident stand at 10·68 per cent. Of the deaths, 89 were caused by cerebral and spinal diseases, 21 by consumption, 8 by bronchitis, 39 by pneumonia, 1 by pleurisy, and 33 by colitis. Of the year's admissions, 62 owed their insanity to such moral causes as domestic trouble, mental anxiety, religion and fright; while of the physical causes hereditary influence and intemperance in drink claim an unenviable prominence, the former accounting for 109 cases, and the latter for 72. Remarking on the alcoholic cases, Dr. Jones says that "women relapse from alcohol and are readmitted with far greater frequency than men. Their weakened inhibition appears to be unable to withstand the slightest temptation," and he further gives his opinion that the "proper and best treatment is that of long detention in inebriate homes, which naturally cannot apply to asylums where patients are discharged when mentally fit." Dr. Jones thinks that consumption is even more frequent in asylums than statistics demonstrate, and he is probably right in this. Colitis, as already said, caused death in 33 cases, and so prevalent was this disease in July that the detached hospital was opened to accommodate the sufferers. We are glad to notice that Dr. Jones has returned to the open-fireplace system of warming the wards, and the artificial system is relegated to its proper and entirely subordinate position. The committee say that "much care and attention are devoted by the medical superintendent to the work of the institution." The weekly cost per head was 11s.

### DORSET COUNTY ASYLUM.

On January 1st, 1899, there were in this asylum 714 patients. Of this total 550 were chargeable to the county, 93 were private patients, and 71 were paupers admitted from districts beyond the county of Dorset. On December 31st the total number resident was 707. There were 96 first admissions, 19 readmissions, 39 recoveries, 6 discharged relieved, 36 not relieved, and 41 deaths. The average daily number resident during the year was 718, and the total number of persons under care was 822. Reckoned on the number of admissions and excluding transfers the recoveries stand at 37·14 per cent., and reckoned on the average number resident the deaths stand at 5·71 per cent., the first of these percentages being about the average and the latter much below it. Cerebral and spinal diseases are responsible for 8 of the deaths, consumption for 2, pneumonia for 2, pleurisy for 1, bronchitis for 4, tuberculosis for 2, and dysentery for 5. It will be seen from the above figures that only 9 patients died of lung diseases during the year, and that only two of these were cases of phthisis. Including the two cases of general tuberculosis we have 4 deaths from tubercular disease; a low mortality which Dr. MacDonald attributes to the open-air life led by the patients and to the splendid site of the asylum. The deaths from dysentery occurred at a time when the asylum was overcrowded and to this, Dr. MacDonald thinks, the disease was due. That this condition will intensify and spread dysentery is likely enough; but we greatly doubt whether the slight overcrowding at the Dorset Asylum (it would hardly get below 1,000 cubic feet of air per patient) would account for the outbreak at an institution where the climate is fine and the open-air treatment so much practised. The truth is that this "asylum colitis" resembles syphilis in one thing. "We don't know who invented it"; but we are entirely opposed to the views of the Commissioners in Lunacy, quoted by Dr. MacDonald on page 13 of this report, that colitis ought to be bracketed with an insanitary condition of the asylum, if by that term be meant a faulty drainage system; and we believe the experience of Dr. MacDonald with the Dorset drainage is that of many other medical superintendents, viz., that no defect could be found, and no connection traced between the disease and the drainage. The fact that the disease vanishes for months, or maybe years, when the drainage has not been touched, would seem to prove this. Our own experience is that climatic conditions constitute one factor, and that the germs may remain latent for a long period in the closets, probably in the woodwork and crevices generally; and that prevention is best assured by the daily use of the strongest safe disinfectants and constant scrubbing of the closets with hot water. If to this could be added strict personal cleanliness on the part of the patient we believe the disease would disappear. The first two conditions are easily enough carried out; but everyone conversant with asylums knows how difficult the last would be to accomplish. Another difficulty presents itself in early isolation of infected cases because the disease often escapes observation for a day or two. With feeble, demented cases this nearly always happens. The wards for private patients have been full throughout the year, a fact which ought to stimulate the committees of other County Asylums to run up blocks for the treatment of such cases. The asylum is lighted by electricity, and the cost per unit was 1¾d. during 1899. The committee say that "Dr. McDonald continues to merit our highest approval." The weekly rate per head for county patients is 8s. 9d.; for out-county patients, 14s.; and private patients pay from 10s. to £2 2s.

